# Rapid monitoring of high‐mannose glycans during cell culture process of therapeutic monoclonal antibodies using lectin affinity chromatography

**DOI:** 10.1002/jssc.202100903

**Published:** 2022-02-03

**Authors:** Jun Kim, Methal Albarghouthi

**Affiliations:** ^1^ AstraZeneca BioPharmaceuticals R&D Gaithersburg Maryland USA

**Keywords:** high mannose, lectin affinity chromatography, process analytical technology, real‐time monitoring, two‐dimensional liquid chromatography

## Abstract

We developed a simple high‐performance liquid chromatography assay to monitor high‐mannose glycans in monoclonal antibodies by monitoring terminal alpha‐mannose as a surrogate marker. Analysis of glycan data of therapeutic monoclonal antibodies by 2‐aminobenzamide assay showed a linear relationship between high mannose and terminal mannose of Fc glycans. Concanavalin A has a strong affinity to alpha‐mannose in glycans of typical therapeutic monoclonal antibodies. To show that terminal mannose binds specifically to Concanavalin A column, exoglycosidase‐treated monoclonal antibodies were serially blended with untreated monoclonal antibodies. Linear responses of terminal‐mannose binding to the column and comparable data trending with high mannose levels by 2‐aminobenzamide assay confirmed that terminal‐mannose levels measured by the Concanavalin A column can be used as a surrogate for the prediction of high‐mannose levels in monoclonal antibodies. The assay offers a simple, fast, and specific capability for the prediction of high‐mannose content in samples compared with traditional glycan profiling by 2‐aminobenzamide or mass spectrometry‐based methods. When the Concanavalin A column was coupled with protein A column for purification of antibodies from cell culture samples in a fully automated two‐dimensional analysis, high‐mannose data could be relayed to the manufacturing team in less than 30 min, allowing near‐real‐time monitoring of high‐mannose levels in the cell culture process.

**Article Related Abbreviations**
2‐AB: 2‐aminobenzamide
CM: cell‐culture medium
Con A: concanavalin A
mAb: monoclonal antibody
MPA: mobile phase A
MPB: mobile phase B
MPC: mobile phase C

## 1 INTRODUCTION

High mannose in therapeutic monoclonal antibodies (mAbs) are critical quality attributes that have an impact on drug clearance in vivo [1, 2, 3, 4, 5, 6]. Although mannose is typically closely monitored and tightly controlled during cell culture processes, existing analytical methods to assess glycan heterogeneity often take too long to provide timely feedback to manufacturing teams. In many cases, due to delays in analysis, supporting analytical data are used only for batch records or as a future reference. With the implementation of continuous manufacturing practices in the pharmaceutical industry in the near future, the ability to provide analytical data to the production team in a short time, if not in real‐time, will take on increased importance [7, 8, 9, 10, 11, 12]. A quick and simple testing method that can be easily performed in the proximity of the production floor would ensure timely corrective action when, for instance, a sudden spike of mannose is detected after the addition of various cell culture feed ingredients in the manufacturing process for therapeutic mAbs.

A high‐throughput, microchip‐based assay for monitoring high mannose plus hybrid‐type glycans versus complex‐type glycans in crude cell culture supernatant samples have been successfully demonstrated [13]. However, due to additional sample preparation with endoglycosidase H (37°C for 2 h), a reliable control may be needed for assay precision.

MS coupled with LC is used for the relative quantitation of glycans, either at the intact level or for site‐specific glycan information at the peptide level after protease digestion [14, 15, 16, 17]. Although it can provide a direct assessment of high‐mannose levels in mAbs, performing mass spectrometry on the manufacturing floor has not been widely implemented due to challenges in operation and maintenance as well as the complexity of a system that often requires specialized training for analysts. Hence, for many years, HPLC‐based assays have been considered the gold standard for process analytical technology platforms to assess product quality attributes on or near the manufacturing floor [18, 19, 20]. Assessment of mannose levels in cell‐cultured mAb samples with traditional glycan quantitation methods by HPLC requires purification of mAbs from other cell components, typically by affinity‐based chromatography (e.g., protein A), followed by the release of glycans by an amidase (e.g., peptide‐*N‐*glycosidase F) and extensive labeling with fluorescent reagents (e.g., 2‐aminobenzoic acid, 2‐aminobenzamide [2‐AB], etc.) and further clean‐up by SPE [21]. The entire process is time‐consuming and often requires precise control during each step to maintain consistent recovery of glycans, which affects the repeatability of data. For these reasons, these methods have been mostly performed in specialized analytical laboratories. To our knowledge, an HPLC assay that can directly measure high‐mannose levels of mAb glycans in cell‐culture medium (CM) in a relatively short time, such as 2 h, has yet to be developed.

Terminal mannose can be quantitated by using a lectin‐based concanavalin A (Con A) column, which is commercially available for use in quality control laboratories. Con A is a lectin protein that is derived from *Canavalia ensiformis* and has a strong affinity to alpha‐mannose, which is present in many mAb glycans. To find an alternative approach to direct high‐mannose quantitation, we explored the use of terminal mannose as a surrogate to measure levels of high mannose. Historical glycan data, acquired by a 2‐AB labeling method and hydrophilic interaction chromatography (HILIC), from three mAbs were analyzed to identify any relationships between levels of high mannose and terminal mannose. Once it was verified that the correlation was linear, we applied the Con A HPLC assay to monitor terminal‐mannose levels in other mAbs to further confirm the linearity between the two glycan species. When the high‐mannose glycan group was considered part of the terminal‐mannose group, it was plausible to assume that a linear relationship exists. Adding an online purification step with the protein A column made it possible to consolidate the multidimensional separation platform on a single instrument and achieve an additional reduction in processing time. Here we present a terminal‐mannose quantitation method that can be used as a platform assay to estimate high‐mannose levels in mAbs and can be executed on manufacturing floors to support near–real‐time data feedback.

## 2 MATERIALS AND METHODS

### 2.1 Samples

All tested mAbs were proprietary, fully humanized immunoglobulin Gs developed by AstraZeneca.

### 2.2 Chemicals and instrumentation

All chemicals and reagents were purchased from Sigma‐Aldrich and Millipore unless otherwise noted.

#### 2.2.1 Columns

Two types of Con A columns were tested. HiTrap Con A‐4B (catalog no. 28952085, 1 mL) was purchased from Cytiva Life Sciences and used for 1‐D separation. A ProSwift Con A‐1S (catalog no. 074148, 5 × 50 mm) monolithic column was purchased from Thermo Fisher Scientific and used for two‐dimensional separation, using a protein A column as the first dimension. Con A, as a tetramer at neutral and alkaline pH, dissociates into active dimers that bind to alpha‐d‐mannopyranosyl (and, with weaker affinity, to alpha‐D‐glucopyranosyl) below pH 5.6. Divalent metal ions (Ca^2+^, Mn^2+^) are needed to keep Con A active for binding, according to the column manufacturer's instructions. Mannose‐rich horseradish peroxidase had been previously tested with a ProSwift Con A‐1S column by the manufacturer to determine its mannose‐specific binding.

A Poros pre‐packed protein A column (catalog no. 2100100, 2.1 × 30 mm) was purchased from Thermo Fisher Scientific.

#### 2.2.2 HPLC conditions

An Agilent 1200 HPLC system equipped with a quaternary pump was used for method development and testing of mAb products by either 1‐D (Con A column only) or 2‐D (protein A–Con A MS/MS) analysis. The column compartment had a pre‐installed two‐position, six‐port valve.

In the 1‐D Con A method, mobile phase A (MPA) was 50 mM sodium acetate, 0.2 M NaCl, 2 mM MnCl_2_, pH 5.3, and mobile phase B (MPB) was 100 mM α‐methyl mannopyranoside (catalog M6882; Sigma‐Aldrich) added to MPA. The flow rate was 0.75 mL/min at 25°C column temperature for 0–4 min with 100% MPA, 4–5 min with 0–80% MPB, 5–10 min with 80% MPB, and 10–18 min with 100% MPA for re‐equilibration. These gradients were optimized a few times during method development.

For 2‐D tandem chromatography, an external two‐position, six‐port valve (catalog no. G1158A; Agilent) was installed to test cell‐cultured mAbs without prior offline purification. The Poros protein‐A column was installed on the first two‐position, six‐port switch valve, and the Con A column was attached on the second two‐position, six‐port switch valve, which was pre‐installed in the column compartment. MPA and MPB were the same as described for the 1‐D Con A method. Mobile phase C (MPC) was 50 mM citric acid, pH 3, which was used to elute protein A–bound mAb products to the Con A column. Protein A binding of samples with MPA and Con A binding with MPC were confirmed before 2‐D method development. Twenty microliters of sample were loaded at a concentration of 2 mg/mL with 100% MPA between 0−2.5 min. Protein A‐bound product was eluted to the Con A column for 0.75 min with 20% MPA and 80% MPC, followed by a wash with 100% MPA for the Con A column between 3.25–10 min. The flow rate was 0.75 mL/min and the column temperature for both columns was 25°C. The valve position for the protein A column was offline between 7.5 and 18 min, and the Con A column was online between 3.5 and 18 min. The Con A column bound product was eluted with 50% MPA and 50% MPB between 10.5 and 15 min. Columns were equilibrated with MPA between 15 and 20 min, which resulted in about 2 min of equilibration for Protein A and 3 min of equilibration for the Con A column. Peaks were detected by measuring UV absorbance at a wavelength of 280 nm.

#### 2.2.3 Enzyme digestion for positive control sample

To verify the binding of terminal mannose to the Con A column, glycans enriched with terminal mannose were generated by treatment with β‐*N*‐acetylhexosaminidase (catalog no. GK80050; Agilent) for use as a positive control for testing mAbs. In 20 μL of each mAb at 10 mg/mL, 20 μL of 5x buffer included in the kit, 4 μL of enzyme, and 56 μL of H_2_O were added. Samples were mixed and incubated at 37°C for 2 h. The enzyme removes terminal, nonreducing β‐*N‐*acetylglucosamine residues from glycans to expose buried alpha‐mannose that binds to the Con A column. By blending this mannose‐exposed species with nontreated glycans and testing by Con A, we confirmed that the Con A column was specific to terminal mannose. With the appropriate control samples, which had previously been verified for levels of terminal mannose and high mannose, the high‐mannose level in the tested samples was estimated.

### 2.3 2‐AB hydrophilic interaction chromatography

mAbs were digested with peptide‐*N‐*glycosidase F in 50 mM Tris HCl buffer, pH 7.8, at 37°C overnight to release glycans from the mAbs. The released glycans were labeled with 2‐AB by reductive amination, and the labeled glycans were then purified using a GlykoClean S Plus cartridge and analyzed by HILIC on an Acquity UHPLC BEH glycan column. The 2‐AB–labeled glycans were detected with a fluorescence detector with excitation at 330 nm and emission at 420 nm.

### 2.4 Historical data comparison

Three mAb products (mAb1, mAb2, and mAb3) were chosen to assess the relationship between levels of terminal mannose and high mannose. Historic product release data tested by 2‐AB‐HILIC were compared. Since high mannose glycans contain mannose at the terminal, they were counted as terminal mannose species that can be measured by the Con A column.

### 2.5 Sample preparation

Samples of positive control treated with β‐*N*‐acetylhexosaminidase and non‐treated drug substance were blended to generate a linear response for percent mannose binding. For direct CM sample testing by 2‐D LC, titers of mAbs in CM collected on various days were adjusted to 2 mg/mL with 20 mM Tris, pH 7.4. Twenty microliters of each prepared sample was injected. Reference standard for each mAb product was included in each sequence to determine system suitability as well as for use as an assay control for peak identification and quantitation. Unless specified, the same peak integration method (e.g., baseline correction mode, slope sensitivity, and peak width) was used for all samples, including a reference standard, in the same sequence.

## 3 RESULTS AND DISCUSSION

**FIGURE 1:**
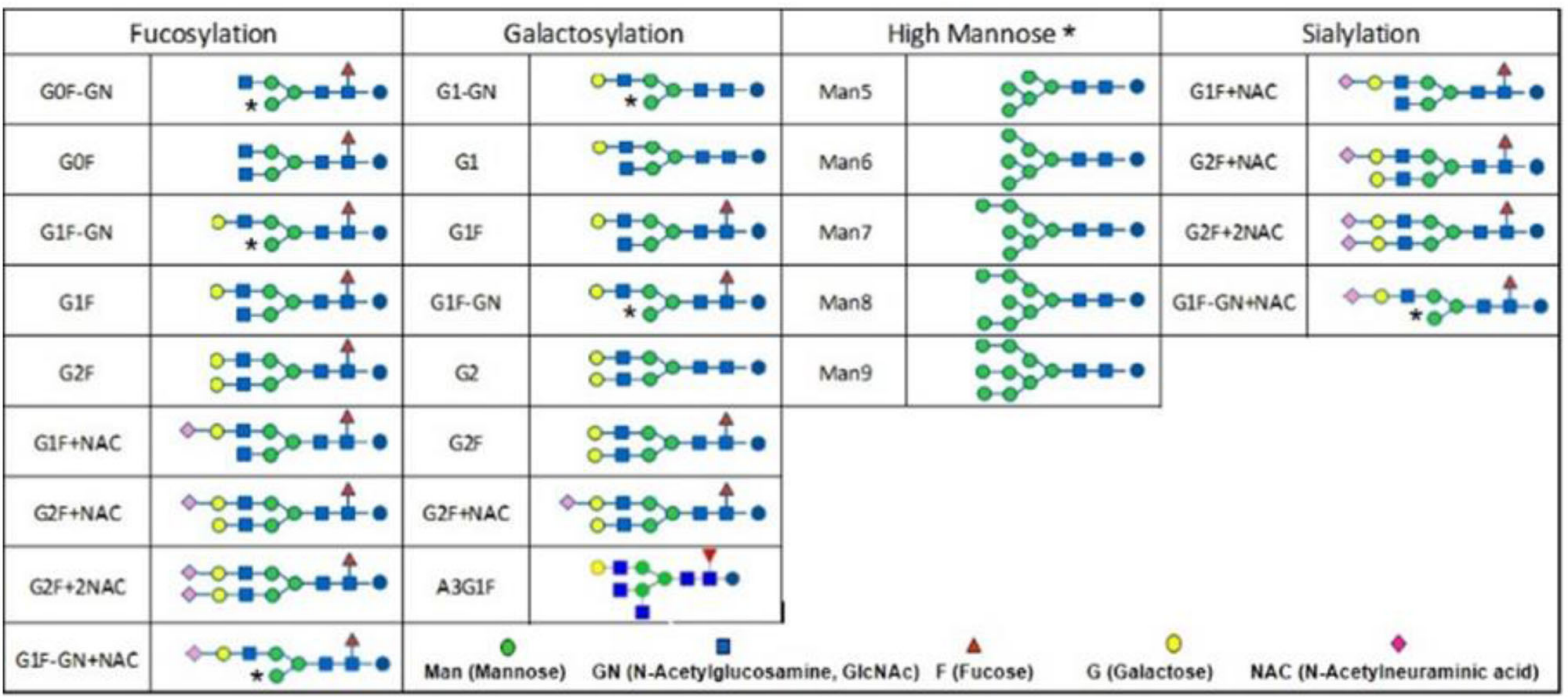
Representative monoclonal antibody (mAb) glycan structures produced in the Chinese hamster ovary (CHO) cell line. Asterisks indicate glycan species with terminal mannose

Typical glycan structures in therapeutic mAbs produced by the Chinese hamster ovary cell line are shown in Figure 1. The NS0 cell line produces additional *N*‐glycolylneuraminic acid (instead of *N*‐acetylneuraminic acid) and galactose‐alpha‐1,3‐galactose. Alpha‐mannose (Figure 1) is exposed in some glycan species and binds to the Con A column. All high‐mannose species contain terminal alpha‐mannose and hence bind to Con A.

### 3.1 Historical data mining

**FIGURE 2:**
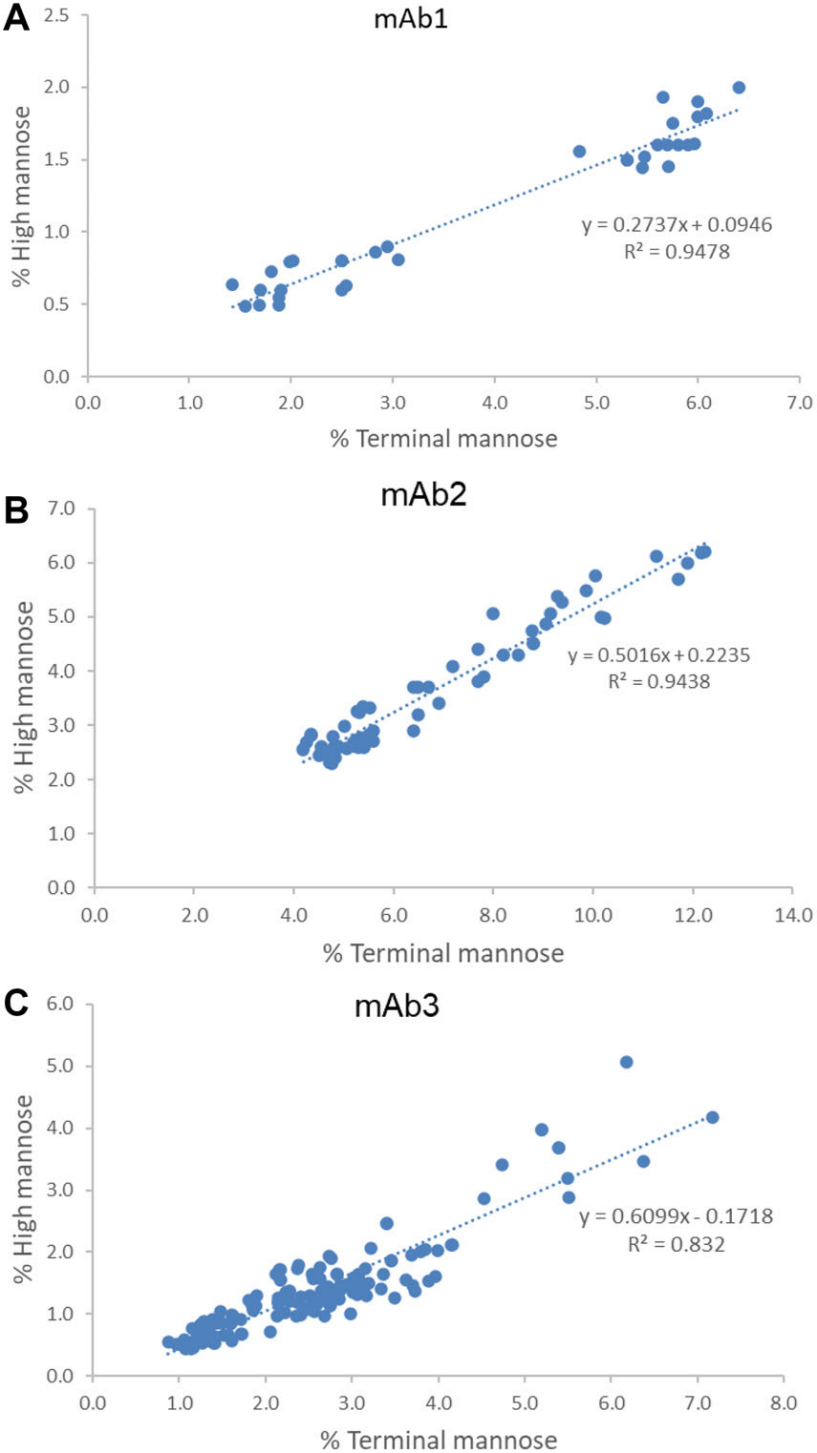
Comparison of terminal mannose and high mannose in monoclonal antibody 1 (mAb1 ) (A), mAb2 (B), and mAb3 (C) tested by 2‐aminobenzamide (2‐AB)–HILIC. The slope indicates ratios between two glycan species

When product release data for mAb1, mAb2, and mAb3 were assessed, the 2‐AB‐HILIC results showed a linear relationship between levels of terminal mannose and high mannose in each product, although the ratios between the two glycans were product specific (Figure 2). All three assessed mAbs were from the Chinese hamster ovary cell line and had levels of high mannose of 0.5–2.0% (mAb1), 2.3–6.2% (mAb2), and 0.4–5.1% (mAb3). Although slight differences in glycan heterogeneity were observed in batch‐to‐batch production, a high‐mannose level was typically maintained from the end of the cell culture process throughout product storage. Our analysis showed that ratios between terminal mannose and high mannose were relatively consistent within the product line (mAb1: *N* = 68, 2.2 < ratio < 4.2, *R* ^2^ = 0.9478; mAb2: *N* = 65, 1.5 < ratio < 2.2, *R* ^2^ = 0.9438; mAb3: *N* = 162, 1.2 < ratio < 3.0, *R* ^2^ = 0.832) (Figure 2). mAb3 showed low levels of high mannose and terminal mannose, which might have contributed to a lower *R* ^2^ value than that seen in the other two mAbs. As shown by the different slopes (0.2737, 0.5016, and 0.6099) of linear curves, different products may have product‐specific ratios between levels of two glycan species.

### 3.2 Con A chromatography

**FIGURE 3:**
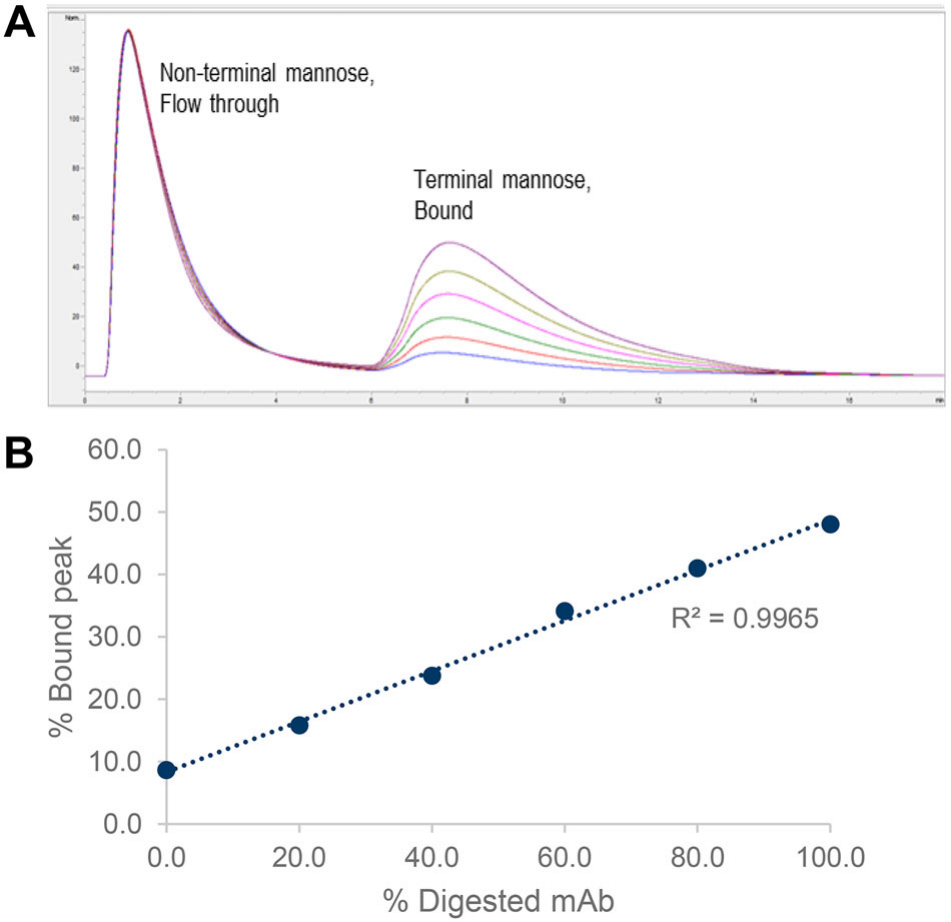
Specificity and linearity of monoclonal antibody (mAb) on the concanavalin A (Con A) column with serially blended samples. mAb with non‐terminal mannose (flow‐through) was clearly separated from mAb with terminal mannose (bound) by the Con A column (A). Percentage bound peak on Con A column showed a linear trend when samples containing 0, 20, 40, 60, 80, and 100% β‐*N*‐acetylhexosaminidase–treated drug substances were injected (B)

To confirm the specificity and linearity of the HiTrap Con A column used to determine terminal mannose, we enriched terminal mannose in mAb4 by β‐*N‐*acetylhexosaminidase and serially blended the treated antibody with the nontreated antibody. β‐*N‐*acetylhexosaminidase removed terminal β‐*N‐*acetylglucosamine, exposing alpha‐mannose, which binds to the Con A column. The results showed a proportional increase between samples containing enriched terminal mannose and bound/eluted alpha‐mannose peak areas on the Con A column (Figure 3).

The broad peak shape of the bound and eluted peak shown in Figure 3 was assumed to be from the glycan heterogeneity of the mAbs, which caused varying binding strength between terminal mannose and the Con A column. This effect poses challenges to peak integration (discussed herein “Data normalization”), and data normalization is proposed to improve assay precision. Intermediate precision was also tested for multiple mAbs, with comparable results among different days or with different instruments (data not shown).

### 3.3 Peak identification

**FIGURE 4:**
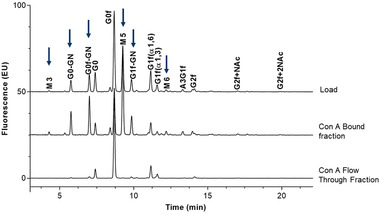
Glycan peak identification of monoclonal antibody (mAb) by 2‐aminobenzamide (2‐AB)–HILIC. Glycan profiles among the load, concanavalin A (Con A)‐bound, and Con A flow‐through fractions were compared. Terminal‐mannose species present in original samples were not present in the flow‐through fractions, indicating that they were bound to the Con A column. Arrows indicate the presence of terminal mannose

Bound and flow‐through fractions were collected from mAb and tested by the 2‐AB–HILIC method for peak identification. Most of the bound fractions contained terminal‐mannose species, and none of the glycans with terminal mannose were found in the flow‐through fraction. High mannose species were greatly enriched in the bound fractions but not detected in the flow‐through fractions as expected (Figure 4).

Glycans are complex structures that do not always contain exposed terminal mannose. A small amount of non‐terminal mannose glycans (including G0f) was observed in the bound fraction, presumably due to the dimeric nature of mAbs, in which one heavy‐chain side contains terminal mannose and the other heavy chain does not.

### 3.4 Estimation of high mannose from Con A chromatography

**FIGURE 5:**
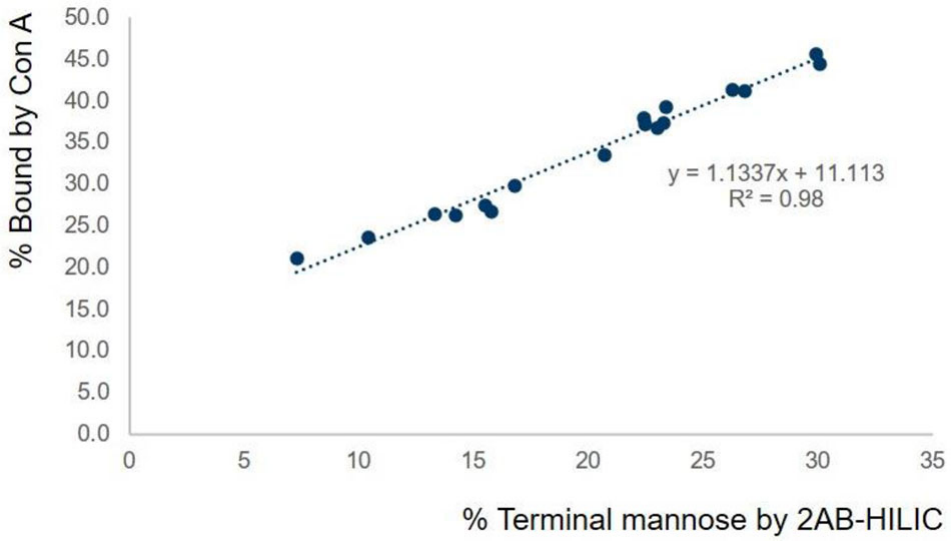
2‐aminobenzamide (2‐AB)–HILIC versus concanavalin A (Con A) for terminal‐mannose assessment for 17 cell‐culture medium (CM) samples purified by offline protein A column

The percentage of terminal mannose by the Con A method was proportional to the percentage of terminal mannose by the 2‐AB–HILIC method for the same mAb. When 17 mAb CM samples were tested by both 2‐AB–HILIC and Con A, the data trend between the two was linear (Figure 5).

Although the data produced by the two methods did not exactly correlate one‐to‐one, once sufficient 2‐AB–HILIC and Con A data were accrued, the percentage of high mannose in specific mAb could be accurately predicted by Con A by using simple data conversion. Table 1 shows the predicted percentage of high mannose by the Con A method after converting the percentage bound by Con A to the percentage of high mannose, using a regression curve from previously collected data (2‐AB–HILIC, percentage of high mannose vs. Con A, percentage bound). The estimated percentage of high mannose by Con A was comparable to the numbers measured by 2‐AB–HILIC, indicating that the Con A method can be useful for simple trending annormalysis of mAb glycans during cell culture. The availability of additional 2‐AB–HILIC and Con A data for mAb products will improve the accuracy of predictions of the percentage of high mannose by Con A.

**TABLE 1:** Conversion to percent high mannose from percent bound by concanavalin A (Con A) by applying conversion factor generated from historic percent bound by Con A to percent high mannose by 2‐AB–HILIC

**Bioreactor condition**	**Collection day**	**% Bound by Con A**	**Estimated % high mannose by Con A**	**% High mannose by 2‐AB–HILIC**	**Difference (%)**
A	8	26.3	4.6	4.7	0.1
	9	36.8	9.9	9.9	0.0
	11	37.3	10.1	11.2	1.1
	14	39.2	11.1	9.8	1.3
B	8	26.4	4.7	4.8	0.1
	9	29.8	6.3	6.5	0.2
	11	44.5	13.8	15.4	1.6
	14	38.0	10.5	11.7	1.2
C	7	23.7	3.3	3.2	0.1
	9	33.5	8.2	7.6	0.6
	11	41.4	12.2	11.4	0.8
	14	45.7	14.4	13.4	1.0
D	7	21.1	2.0	2.3	0.3
	9	27.4	5.2	5.1	0.1
	11	37.4	10.2	9.2	1.0
	14	41.3	12.1	10.8	1.3

Abbreviations: 2‐AB, 2‐aminobenzamide; Con A, concanavalin A.

### 3.5 Data normalization

Various types of glycans with terminal mannose bind to the Con A column, creating different levels of binding strength to the column and resulting in broader peaks during elution. Due to this limitation, some of the mAbs showed less than optimal peak shapes, making consistent integration difficult. Although the method showed high intra‐sequence precision, the peak shape was not always repeatable and required some data normalization. To improve inter‐sequence data precision, an assay control (e.g., reference standard) was included to calculate the relative percentage of binding. The same integration parameters were applied to all samples and controls in the same sequence. A similar data normalization approach has been implemented in other assays, such as potency assays and surface plasmon resonance binding assays, where relative values to reference standards are often reported. Absolute values in these assays are not so consistent in intermediate precision runs, but values relative to assay control often show high levels of precision. Table 2 shows the results of the Con A method to monitor protein A–purified mAb. When data were normalized (Table 2), data trending was more accurate and the relative standard deviation was lower among replicate runs on three consecutive days.

**TABLE 2:** Normalized data to improve precision

**Collection day**	**Without data normalization(% bound by Con A)**				**With data normalization against RS (relative to % RS bound by Con A)**			
	**Day 1**	**Day 2**	**Day 3**	**RSD**	**Day 1**	**Day 2**	**Day 3**	**RSD**
7	49.3	33.3	31.3	26.0	1.0	0.9	0.9	6.2
9	63.2	52.2	50.7	12.3	1.3	1.4	1.4	4.2
11	72.3	62.9	61.2	9.1	1.5	1.6	1.7	6.3
14	70.4	61.4	59.8	8.9	1.5	1.6	1.6	3.7

Abbreviations: Con A, concanavalin A; RS, reference standard; RSD, relative standard deviation (RSD = standard deviation x 100 /average). Relative to % RS bound on Con A = % bound by Con A/% RS bound by Con A.

### 3.6 2‐D protein A– Con A chromatography for monitoring high mannose of mAb in CM

**FIGURE 6:**
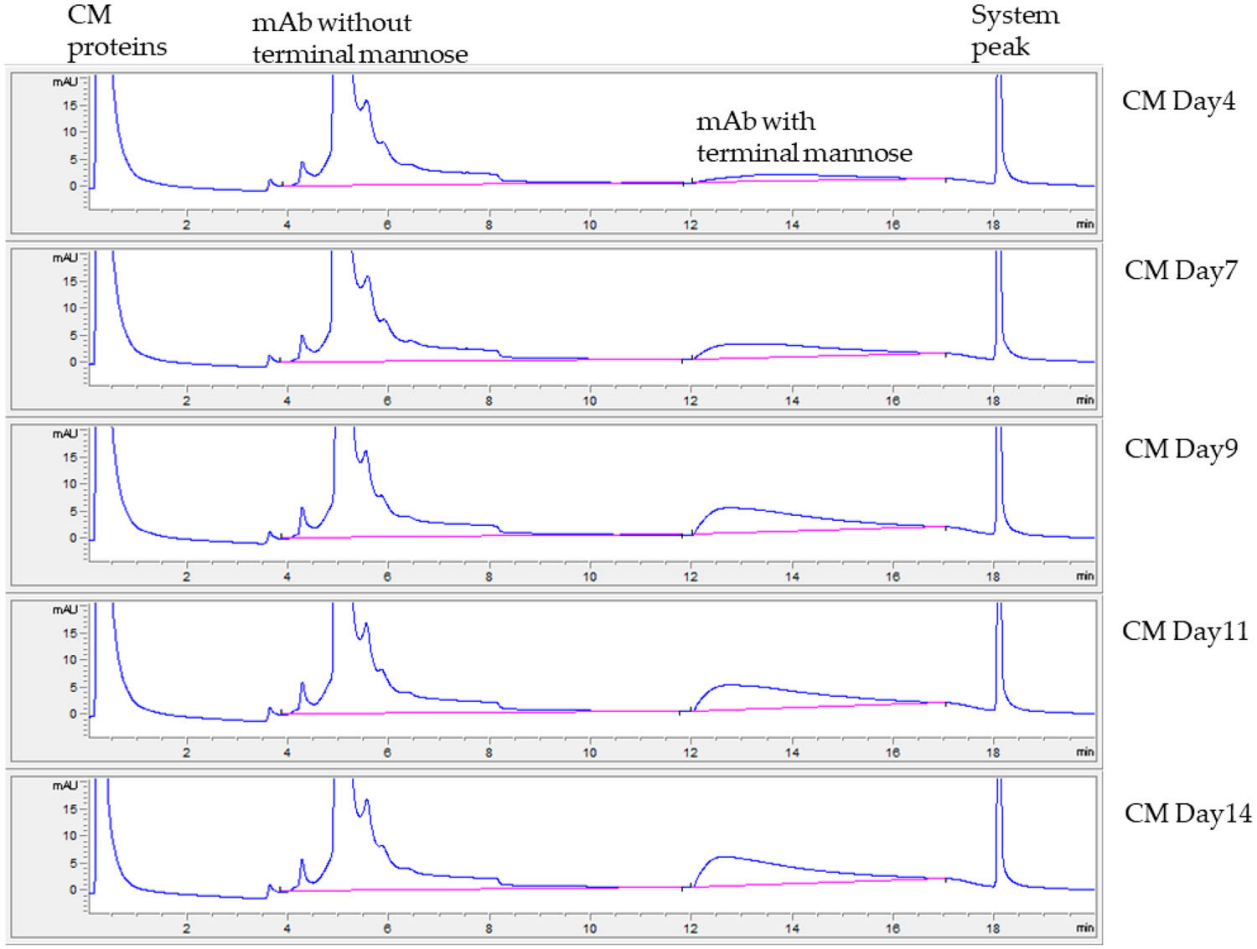
Monitoring of terminal mannose in monoclonal antibody (mAb) cell‐culture medium (CM) collected on 5 different days by 2‐D protein A–concanavalin A (Con A) method on traditional HPLC. Peak integration lines are indicated in pink. Increases in terminal‐mannose peaks when cell culture progresses were visible and quantitated to predict the high‐mannose level of mAb in CM. mAb peak shapes were slightly different than those in Figure 3 due to the use of ProSwift Con A‐1S column with a higher system pressure limit, instead of the Con A‐4B column used in Figure 3

**FIGURE 7:**
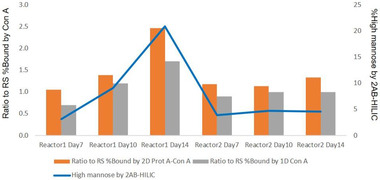
Comparison of glycan assessment among 2‐D protein A–concanavalin A (Con A) method, 1‐D Con A method, and 2‐AB–HILIC for two sets of monoclonal antibody (mAb) cell‐culture medium (CM) samples. Trends in glycan heterogeneity (terminal‐ and high‐mannose levels) were comparable among three methods

To enable real‐time monitoring of high mannose, we developed a fully automated 2‐D chromatography consisting of protein A followed by a Con A column. Simply by adding an additional switch valve, 2‐D separation was possible on a traditional HPLC system by protein A–Con A tandem column chromatography. We tested CM samples with an in‐house–developed 2‐D LC system (Figure 6). The protein A–Con A profiles and results for six CM samples were comparable to those obtained with the 1‐D Con A system (Figure 7), as well as the trend observed with the 2‐AB–HILIC method.

## 4 CONCLUDING REMARKS

The availability of product‐specific information on high‐mannose and terminal‐mannose levels makes it possible to predict high‐mannose levels in mAbs by performing the Con A method with HPLC. Implementation of a method to monitor high‐mannose levels in real‐time during the cell culture process will greatly improve the ability to control a critical quality attribute known to be sensitive to cell culture conditions. Production engineers can perform corrective actions quickly in the event of any adverse incidents to maintain consistent product quality during production.

The 2‐D separation tool will greatly improve the real‐time monitoring capability of CM samples by bypassing offline protein A purification. The Con A method is a simple and fast alternative to other analytical methods to estimate levels of high mannose in CM samples. For example, testing of four CM samples (including the reference standard and the samples shown in Table 2) was completed and the results reported within 2 h. By comparison, the 2‐AB–HILIC method and the MS method take longer to run and may not be practical to be implemented near the bioreactor, thus requiring additional time and resources to manage the logistics of shipping samples for testing at a remote testing site.

When additional data confirm that the glycan heterogeneity of a given product is similar from batch to batch, it will be possible to make accurate predictions of high‐mannose levels by measuring terminal‐mannose levels. Although our study was based on several mAb products, the ratios between these two glycan species in mAb products appeared to be product‐specific and within tight ranges. Although the Con A method may not provide comprehensive information on glycan heterogeneity in mAbs, it can provide a quick assessment of high‐mannose levels, which is often considered a critical quality attribute of therapeutic mAb products. Because many cell culture laboratories may already have traditional HPLC systems and adequately trained analysts to perform LC‐based testing, real‐time monitoring of high‐mannose levels of mAbs in CM can be achieved on the manufacturing floor with the methods presented here.

## CONFLICT OF INTEREST

The authors are employees of AstraZeneca and may hold stock ownership or stock interests in the company.
